# Epigenetic regulation of inflammation in post-operative organ dysfunction: A scoping review protocol

**DOI:** 10.1371/journal.pone.0320829

**Published:** 2025-10-30

**Authors:** Ruairí Wilson, Charlotte Fern, Carl Goodyear, Ben Shelley

**Affiliations:** 1 Anaesthesia, Perioperative Medicine and Critical Care Research Group, School of Medicine, University of Glasgow, Glasgow, Scotland, United Kingdom; 2 School of Infection and Immunity, University of Glasgow, Glasgow, Scotland, United Kingdom; 3 Department of Anaesthesia, Golden Jubilee National Hospital, Clydebank, Scotland, United Kingdom; Xiamen University - Malaysia Campus: Xiamen University - Malaysia, MALAYSIA

## Abstract

**Introduction:**

The inflammatory response to surgery is complex, dynamic and exhibits variability in magnitude and duration among patients undergoing similar operations. Dysregulated inflammation is associated with post-operative complications such as organ dysfunction, particularly after major surgery. Epigenetic modifications enhance (or suppress) selective gene transcription without altering DNA sequences, effectively regulating gene expression. Several studies have investigated epigenetic regulation of the immune system in the context of surgery, often studying organ-specific dysfunction.

**Objectives:**

We propose a novel scoping review protocol to collate and synthesise existing studies investigating epigenetic regulation of post-operative inflammation, as a key mechanism of post-operative organ dysfunction and complications. We will map knowledge gaps to inform future research in this emerging field.

**Methods and analysis:**

This scoping review protocol has been created following the Joanna Brigg’s Institute (JBI) updated guidelines for conducting scoping reviews. The protocol has been further examined alongside the Preferred Reporting Items for Systematic reviews and Meta-Analyses (PRISMA) extension for Scoping Reviews (PRISMA-ScR) checklist and is registered on Open Science Framework (doi.org/10.17605/OSF.IO/CE8FB). Published human studies from 1946 to the present will be considered. Studies will include patients undergoing surgery, where epigenetic regulation of the immune system is investigated alongside assessment of organ dysfunction or complications. Searches will be conducted using Medline (via OVID) and Embase. Two reviewers will independently screen titles, abstracts and full texts of studies meeting the inclusion criteria. Following study screening, a customised data extraction form will collect study information related to the review questions and inclusion criteria (population, concept, context). Results will be presented by diagrammatic mapping of studies and tabular representation of findings.

## Introduction

Post-operative complications are a major cause of morbidity, mortality and increased healthcare cost in patients undergoing surgery [1–3]. The ‘stress response’ to surgical trauma results from well-choreographed activation of autonomic, neuroendocrine, metabolic, and inflammatory responses, necessary to maintain host homeostasis and facilitate tissue repair [4]. Unchecked inflammation can however have detrimental effects on capillary permeability, immune function, wound healing and organ function, culminating in post-operative complications. Serological markers of inflammation have identified that the magnitude of the post-operative inflammatory response is associated with post-operative complications following surgery [5–7]. The degree of post-operative inflammation is associated with multi-organ morbidities, including: myocardial injury [8], lung injury [9], post-operative cognitive dysfunction [10] and acute kidney injury [11].

A ‘genomic storm’ occurs within hours of major surgery, affecting more than 80% of cellular pathways, dynamically altering the leucocyte transcriptome, with upregulation of the innate immune system and suppression of adaptive immune responses [3,12]. In patients undergoing major thoracoabdominal surgery, Allen et al demonstrated differential expression of 522 genes governing leukocyte function 24-hours after surgery; 248 (48%) associated with innate inflammation were upregulated while 274 (52%) associated with adaptive immunity were downregulated [12]. In this study, within-patient gene expression change was associated with postoperative infection, hospital length of stay, and outcomes were worse in patients in whom post-operative gene expression was most radically altered.

The potential for host genomics to impact peri-operative outcomes has received some study, and whilst it has been possible to identify single nucleotide polymorphisms which might lead to specific perioperative morbidity syndromes (e.g., butyrylcholinesterase deficiency [13]), it is unlikely that polymorphisms are responsible for the wide heterogeneity seen in responses to surgery. The value of studying genetic susceptibility to complications is limited by the static nature of the genome; should predictors of outcome be identified, these genetic changes are fixed, with limited potential for therapeutic intervention. By contrast, the epigenome is dynamic and amenable to therapeutic manipulation or upstream modification. Future epigenetic study therefore has potential to predict and modify perioperative risk.

### Epigenetics and inflammation

Epigenetics concerns the study of how cells regulate gene activity without changing the DNA sequence [14]. Cellular DNA is bound in chromatin as a complex of DNA and proteins. Modification of these proteins (classically by DNA methylation, histone modification or small non-coding RNAs (such as microRNAs)) enables differential gene expression, changing the way a cell responds to a stimulus. Classical descriptions of the immune system describe the innate and adaptive responses, with only the adaptive immune response able to build immunological memory. In recent years, epigenetic reprogramming of innate immune cells in response to stimulation has been recognised [15,16]. Following an initial challenge from an inflammatory insult, cells return to a non-activated state, but through modification to the epigenome, are primed for response to a subsequent stimulus. After a secondary challenge, rapid and enhanced recruitment of transcription factors escalate the pro-inflammatory phenotype.

### Epigenetics, inflammation and post-operative complications

Investigation of how epigenetic mechanisms regulate the post-operative inflammatory response has evolved within the last decade, from whole-genome methylation profiling to analysis of specific histone methylation signatures and more recently to the study of microRNAs. The range of surgical specialties investigating epigenetic regulation of inflammation has increased, as has the scope of assessed complications. For example, several studies investigate epigenetic mechanisms of post-operative delirium [17–21], most commonly in the context of neurosurgery. Wahba and colleagues investigated genome-wide DNA methylation profiles in 40 patients undergoing neurosurgery, 11 of whom developed post-operative delirium [19]. While the study was exploratory, DNA methylation at certain CpG sites suggested a link between glial cell differentiation and systemic inflammation pathways in the development of delirium after neurosurgery. Additional studies led by Yamanashi in neurosurgical patients further implicate DNA methylation at specific CpG sites as an epigenetic inflammatory mechanism contributing to the development of post-operative delirium [20,21].

In cardiac surgery, Fischer and colleagues have identified DNA methylation loci associated with the immune response to surgical tissue injury and post-operative atrial fibrillation [22], a common complication in this patient cohort. Laudanski and colleagues initially investigated PU.1 gene promoter methylation (a key transcriptional regulator of monocyte colony stimulating factor) in 20 patients undergoing surgery with cardiopulmonary bypass [23], but did not assess organ dysfunction. This group then conducted a larger study of 59 patients undergoing cardiac surgery, investigating specific methylation (H3K4me3) and acetylation (H3K27ac) patterns on inflammatory genes, demonstrating prolonged serological histone release and inflammation, persisting 3-months after surgery [24].

In an elderly population undergoing major colorectal and orthopaedic surgery, Sadahiro and colleagues reported DNA methylation as a key mechanism influencing inflammation after surgery, describing rapid post-operative methylation, with stable expression 7-days post-operatively [25]. Recently, Albers-Warlé and colleagues demonstrated dynamic epigenetic modification of chromatin structures in key inflammatory genes before and after major colorectal surgery and the significant effect this has on post-operative cytokine production, indicating a fundamental role for epigenetics in regulating the inflammatory response to surgery [26].

The number of studies investigating epigenetic regulation of inflammation and post-operative complications has increased in the last decade, however research remains largely limited to a small number of observational studies. To our knowledge, only one systematic review investigates epigenetics in the perioperative period and it focuses on microRNAs as prognostic biomarkers of post-operative atrial fibrillation [27]; none of the 6 included studies investigate epigenetic-regulated inflammation as a pathogenic mechanism of post-operative atrial fibrillation [28–33]. No published scoping reviews exist on this subject and comprehensive narrative reviews on epigenetics in the perioperative period are now at least a decade old [34,35]. This scoping review protocol outlines an appropriate methodology to systematically collate, synthesise and explore the key themes of recent studies investigating the epigenetics of perioperative inflammation and will highlight research gaps, assisting future research design in this promising and expanding area.

## Aim

To identify and map existing evidence of inflammatory post-operative organ dysfunction (and complications) mediated through epigenetic regulation.

In keeping with the Joanna Brigg’s Institute (JBI) updated guidelines for scoping reviews [36–38], we will systematically:

- identify types of evidence available;
- identify and analyse knowledge gaps;
- determine key concepts and definitions (for example, how epigenetic modifications are defined and described in the literature);
- examine how research is conducted;
- identify key factors related to epigenetic-regulated inflammation in the context of surgery.

## Review Questions

### Primary question

1. What evidence exists for epigenetic-regulated inflammation as a mechanism of organ dysfunction after surgery?

### Secondary questions

2a: What types of studies are available?

2b: How are epigenetic modifications defined and described in the literature?

2c: What specific acute organ dysfunctions (or other complications of surgery) have been studied?

2d: What types of surgery have been studied?

2e: Where are the knowledge gaps in the field?

## Methods

This scoping review protocol has been created following guidance from the updated JBI guidelines for conducting scoping reviews [36,37] and the Arskey and O’Malley framework [39]. The protocol has been further examined alongside a completed Preferred Reporting Items for Systematic reviews and Meta-Analyses (PRISMA) Extension for Scoping Reviews (PRISMA-ScR) checklist [40] (S1 Fig). A scoping review is appropriate for our exploratory review questions, which aim to systematically identify, collate and map available evidence pertinent to our research questions and inclusion criteria.

### Inclusion criteria

This scoping review will include all available human studies which investigate acute organ dysfunction or complications (occurring within 3 months of surgery) resulting from epigenetic mechanisms of inflammation in the context of surgery (Table 1).

**Table 1:** Inclusion and exclusion criteria.

	Inclusion criteria:	Exclusion criteria:
**Population:**	Adult humans undergoing surgery (including any surgical specialty, elective (planned) or emergency (unplanned) surgery).	1. Animal studies. 2. Studies investigating outcomes after transplant surgery.
**Concept:**	Inflammation regulated by epigenetic mechanisms (including DNA methylation, histone modification and microRNAs).	Studies not investigating inflammation as a mechanism of organ dysfunction.
**Context:**	Acute organ dysfunction (or complications) identified after surgery.	
**Sources:**	Original research, including case reports and case series. English language 1946–2025.	Review articles.

Inclusion and exclusion criteria have been formatted using the JBI-recommended Population, Concept, Context (PCC) framework.

#### Population.

Adult humans undergoing any type of surgery, including unplanned emergency or planned elective operations.

#### Concept.

Inflammation regulated by epigenetic mechanisms. Epigenetic modification typically occurs through DNA methylation, histone modification, or microRNA mechanisms. One sub-question for this review (2b) seeks to address the heterogeneity of terms used to describe epigenetic modifications.

#### Context.

Acute organ dysfunction (or complications) identified after surgery. We anticipate several studies will focus investigation on a particular organ dysfunction (for example, post-operative delirium) while other studies will assess for a range of possible post-operative complications. We have limited inclusion to acute complications occurring within 3-months of surgery as the focus of our study is epigenetic regulation of acute post-operative inflammation and the epigenetic regulation of chronic complications of surgery is beyond the scope of this review and risks diluting the research focus and utility of our findings.

### Exclusion criteria

Animal studies will be excluded owing to concerns of translatability and relevance to human organ dysfunction in the context of surgery. Similarly, studies investigating outcomes after transplant surgery will not be included owing to the potent immunosuppressants used in this population which reduces translatability to other patient populations, particularly in the context of post-operative inflammation. Review articles will be excluded given the need to collate and map original research.

### Anticipated methodological challenges

Inflammation, epigenetics and organ dysfunction (or complications) are broad terms which can be heterogeneously defined. Consistency in the application of the described inclusion and exclusion criteria is essential for the conduct of a robust scoping review with minimal bias. For this study, we will include all studies which measure any epigenetic mechanism, alongside a serological measure of inflammation and any acute organ dysfunction or complication occurring after surgery within a time-period of 3-months. While this approach may seem broad and ambitious, the low threshold for inclusion minimizes the risk of bias and our pilot searches strongly indicate a low yield of studies which satisfy these 3 inclusion criteria, favouring feasibility.

We expect a range of studies which measure diverse epigenetic mechanisms and inflammatory processes, described using varying laboratory methods. While this will limit direct comparability, our experience in reviewing the literature strongly suggests that there is such heterogeneity among studies that direct comparability is likely to be challenging due to other non-controlled factors. Furthermore, a principal aim in conducting this study is to map available evidence and to provide insight to guide future research design.

### Databases

Searches will be conducted in Medline (via OVID) and Embase.

### Search strategy

The search strategy aims to locate published studies using the JBI three-step approach [36]. An initial pilot search of Medline (via OVID) and Embase will be undertaken to identify relevant articles. Keywords and index terms used in the title and abstracts of relevant papers will be used to generate a full search strategy for Medline (such as the pilot search in S1 Table) and Embase (S2 Table). The reference list of all included studies will be screened for additional studies. Studies published in English from 1946 to present day will be included for screening. The citation manager software Endnote 21.4 will be used to remove duplicate articles and the online software tool Covidence (Covidence systematic review software, Veritas Health Innovation, Melbourne, Australia) will be used to apply inclusion and exclusion criteria (Table 1) for selection of articles during screening.

### Study selection

At least two independent reviewers will conduct literature screening of titles and abstracts using the inclusion and exclusion criteria. Selected articles will undergo secondary screening of full-text articles prior to data extraction. Reviewers will meet regularly to resolve disagreements through discussion of the article in question against the inclusion and exclusion criteria. Where discussion cannot resolve disagreement, an additional reviewer will assist until consensus is reached. The systematic literature search will be presented in the format of a PRISMA-ScR flow chart for reproducibility and transparency [40]. Results will be reported using descriptive statistics, diagrammatic figures or tables of information, in addition to narrative descriptions in keeping with the JBI guidelines [38].

### Data extraction

Data extraction will similarly be performed by at least two independent reviewers, followed by cross-checking of included articles. Disagreements will be resolved through discussion and mutual agreement of the article in question. Where this fails to resolve a disagreement, consultation with an additional independent reviewer will be undertaken until mutual agreement. Data extraction will include basic descriptive data of each article and information relating to the review question and inclusion criteria. A draft table detailing anticipated data extraction is shown in Table 2, and includes population, concept and context subheadings of the inclusion criteria, as recommended by the JBI [37,38]. The draft data extraction tool will be piloted on 20% of the articles screened for inclusion to test for feasibility and utility in answering the review questions. We anticipate that the data extraction tool may be amended during the review process to enhance our ability to answer the research questions. Disclosure and justification for modifications will be detailed in the subsequent scoping review report.

**Table 2:** Anticipated data extraction table.

Source of evidence (citation):	**Population** Surgical specialty investigated	**Population** Number of patients in study	**Population** Emergency or elective surgery?	**Concept** Organ dysfunction(s) or complication(s) assessed	**Context** Epigenetic modification(s) assessed	**Context** Component of the immune system assessed	**Results** Main fundings of the study

Columns contain basic descriptive data, population-, concept- and context-specific study features related to the research questions and inclusion criteria.

### Data analysis

Descriptive data analyses including frequency analysis of studies will be presented. We anticipate this will involve diagrammatic mapping of studies and tabular representation of findings. Diagrammatic representation is likely to be of particular importance to demonstrate the different types of surgery investigated, organ dysfunctions investigated, and the population sizes recruited to each study. Quality assessment of studies will not be performed as this is beyond the remit of a scoping review.

### Protocol deviations

We aim to conduct our scoping review process in-line with the principles and steps outlined, in keeping with best-practice guidelines [37,38]. However, given the iterative nature of the scoping review process, protocol deviation may be necessary during the search strategy, data extraction or data presentation stages. Disclosure and justification for deviations to the described protocol will be detailed in the subsequent scoping review report.

## Conclusions

This scoping review proposes a novel and systematic approach to collate existing studies on an important and expanding research area. The broad inclusion of diverse study types, including different types of surgery, assessment of a range of post-operative complications, inclusion of multiple components of the immune system and the wide range of described epigenetic modifications is ambitious and has strengths and limitations.Strengths of this approach include a comprehensive assessment of the evolution of research design in this emerging field, identifying themes in recent studies and highlighting gaps in the field (such as under-represented surgical specialties and under-investigated components of the epigenome, immune system and post-operative complications). Limitations in the broad scope of this review include the possibility of reduced generalizability and restriction of included articles to English language. This broad and explorative research question is however appropriate for a scoping review and aligns with our aims to inform on key research themes and gaps in the field to guide future research design.

## Supporting information

S1 Fig Preferred Reporting Items for Systematic reviews and Meta-Analyses extension for Scoping Reviews (PRISMA-ScR) Checklist. (PDF)

S1 Table Table showing a pilot search strategy for the Medline (via OVID) database. (DOCX)

S2 Table Table showing a pilot search strategy for the EMBASE (via OVID) database. (DOCX)
