# Supplementary material for: Epigenetic regulation of inflammation in post-operative organ dysfunction: A scoping review protocol
Source: PLoS One. 2025 Oct 30;20(10):e0320829. doi: 10.1371/journal.pone.0320829 (PMC12574856; doi:10.1371/journal.pone.0320829)
Supplement: S1 Table — (DOCX) [file pone.0320829.s002.docx]

| **Database:** | **Search strategy:** |
| --- | --- |
| Medline (via OVID) | Ovid MEDLINE(R) ALL <1946 to September 23, 2025>  1 surgery/ 41667  2 anaesthesia/ 236  3 anesthesia/ 68326  4 anesthesiology/ 34256  5 postop*.mp. 1109290  6 post-op*.mp. 112082  7 after surgery.mp. 216519  8 surgical.mp. 1756716  9 epigen*.mp. 157541  10 genetic.mp. 2072048  11 dna meth*.mp. 103154  12 histone mod*.mp. 19224  13 histon*.mp. 153811  14 h3k4*.mp. 7692  15 h3k27*.mp. 10625  16 microRNA*.mp. 184341  17 miRNA*.mp. 116621  18 or/1-8 2597789  19 or/9-17 2417848  20 organ d*sfunction.mp. 21107  21 organ injur*.mp. 7686  22 organ failure.mp. 38128  23 complicat*.mp. 4017061  24 postoperative complication*.mp. 491054  25 adverse event*.mp. 278119  26 adverse effect*.mp. 2321169  27 morbidity.mp. 525479  28 mortality.mp. 1594652  29 respiratory failure.mp. 47101  30 hypox*.mp. 260787  31 acute respiratory distress syndrome.mp. 26731  32 pneumonia.mp. 248136  33 shock.mp. 283594  34 hypotension.mp. 81576  35 arrhyth*.mp. 181785  36 myocard*.mp. 670352  37 ischaem*.mp. 72808  38 ischem*.mp. 495146  39 lactate.mp. 161667  40 cardiac death.mp. 34441  41 cardiac arrest.mp. 51647  42 pulmonary embolism.mp. 67298  43 deep venous thrombosis.mp. 14069  44 atrial fibrillation.mp. 122896  45 coma.mp. 54991  46 delirium.mp. 28431  47 confus*.mp. 80175  48 stroke.mp. 428617  49 cerebrovascular accident.mp. 6166  50 pain.mp. 990927  51 kidney injury.mp. 91752  52 kidney failure.mp. 116782  53 renal injury.mp. 15712  54 renal failure.mp. 101356  55 kidney dys*.mp. 4673  56 renal dys*.mp. 25289  57 acute kidney injury.mp. 83332  58 anaem*.mp. 42664  59 anem*.mp. 229857  60 neutro*.mp. 318803  61 leuko*.mp. 435937  62 lympho*.mp. 1214380  63 thrombo*.mp. 562371  64 platel*.mp. 343515  65 pancyto*.mp. 12019  66 marrow suppres*.mp. 2962  67 myelo*.mp. 401808  68 coagulop*.mp. 22095  69 disseminated intravascular coag*.mp. 18867  70 liver failure.mp. 34264  71 liver dys*.mp. 11275  72 hepatic fail*.mp. 10071  73 hepatic dys*.mp. 5825  74 fever.mp. 280224  75 febrile.mp. 46527  76 infect*.mp. 3022696  77 systemic inflammatory response syndrome.mp. 12514  78 sequential organ failure assessment.mp. 6638  79 apache.mp. 15514  80 multiple organ dysfunction score.mp. 207  81 clavien-dindo.mp. 8826  82 surgical site infection.mp. 14437  83 critical care.mp. 100101  84 critical illness.mp. 52058  85 inflam*.mp. 1574004  86 immun*.mp. 4311862  87 c-reactive protein.mp. 117715  88 interleukin*.mp. 450453  89 tumor necrosis factor.mp. 244601  90 exp human/ 22970056  91 review/ 3388871  92 or/20-84 12795794  93 or/85-89 5429864  94 (18 and 19 and 90 and 92 and 93) not 91 2271  95 limit 94 to english language 2125 |

**S1 Table: Table showing a pilot search strategy for the Medline (via OVID) database.**
