# Supplementary material for: Epigenetic regulation of inflammation in post-operative organ dysfunction: A scoping review protocol
Source: PLoS One. 2025 Oct 30;20(10):e0320829. doi: 10.1371/journal.pone.0320829 (PMC12574856; doi:10.1371/journal.pone.0320829)
Supplement: S2 Table — (DOCX) [file pone.0320829.s003.docx]

| **Database:** | **Search strategy:** |
| --- | --- |
| Embase (via OVID) | Embase 1947-Present <1947 to September 23, 2025>  1 surgery/ 1133266  2 anaesthesia/ 171165  3 anesthesia/ 171165  4 anesthesiology/ 27046  5 postop*.mp. 1612652  6 post-op*.mp. 252532  7 after surgery.mp. 322367  8 surgical.mp. 2466835  9 epigen*.mp. 233741  10 genetic.mp. 2937941  11 dna meth*.mp. 160333  12 histone mod*.mp. 35381  13 histon*.mp. 219828  14 h3k4*.mp. 11147  15 h3k27*.mp. 16787  16 microRNA*.mp. 281010  17 miRNA*.mp. 150886  18 or/1-8 4179673  19 or/9-17 3422434  20 organ d*sfunction.mp. 32029  21 organ injur*.mp. 18907  22 organ failure.mp. 104040  23 complicat*.mp. 4408326  24 postoperative complication*.mp. 549624  25 adverse event*.mp. 601432  26 adverse effect*.mp. 333718  27 morbidity.mp. 958040  28 mortality.mp. 2320500  29 respiratory failure.mp. 161266  30 hypox*.mp. 440457  31 acute respiratory distress syndrome.mp. 76327  32 pneumonia.mp. 499607  33 shock.mp. 484433  34 hypotension.mp. 237072  35 arrhyth*.mp. 320636  36 myocard*.mp. 797529  37 ischaem*.mp. 113368  38 ischem*.mp. 1003356  39 lactate.mp. 319855  40 cardiac death.mp. 74578  41 cardiac arrest.mp. 90810  42 pulmonary embolism.mp. 83173  43 deep venous thrombosis.mp. 22078  44 atrial fibrillation.mp. 281598  45 coma.mp. 115401  46 delirium.mp. 60495  47 confus*.mp. 153152  48 stroke.mp. 629354  49 cerebrovascular accident.mp. 491672  50 pain.mp. 1924497  51 kidney injury.mp. 147113  52 kidney failure.mp. 559301  53 renal injury.mp. 24244  54 renal failure.mp. 165437  55 kidney dys*.mp. 39146  56 renal dys*.mp. 42065  57 acute kidney injury.mp. 85283  58 anaem*.mp. 82867  59 anem*.mp. 555382  60 neutro*.mp. 648703  61 leuko*.mp. 865973  62 lympho*.mp. 2119419  63 thrombo*.mp. 1317960  64 platel*.mp. 571125  65 pancyto*.mp. 33759  66 marrow suppres*.mp. 37818  67 myelo*.mp. 725073  68 coagulop*.mp. 38539  69 disseminated intravascular coag*.mp. 18784  70 liver failure.mp. 86846  71 liver dys*.mp. 43328  72 hepatic fail*.mp. 16914  73 hepatic dys*.mp. 9311  74 fever.mp. 637312  75 febrile.mp. 114870  76 infect*.mp. 4189084  77 systemic inflammatory response syndrome.mp. 22302  78 sequential organ failure assessment.mp. 25272  79 apache.mp. 35870  80 multiple organ dysfunction score.mp. 344  81 clavien-dindo.mp. 18341  82 surgical site infection.mp. 22631  83 critical care.mp. 89914  84 critical illness.mp. 51677  85 inflam*.mp. 2430242  86 immun*.mp. 6466915  87 c-reactive protein.mp. 336533  88 interleukin*.mp. 1032092  89 tumor necrosis factor.mp. 623918  90 exp human/ 30394072  91 review/ 3268384  92 or/20-84 16886065  93 or/85-89 8212671  94 (18 and 19 and 90 and 92 and 93) not 91 14906  95 limit 94 to english language 14579 |

**S2 Table: Table showing a pilot search strategy for the Embase (via OVID) database.**
